# Polyhydroxyalkanoate (PHA) Production in *Pseudomonas* sp. phDV1 Strain Grown on Phenol as Carbon Sources

**DOI:** 10.3390/microorganisms9081636

**Published:** 2021-07-30

**Authors:** Iliana Kanavaki, Athina Drakonaki, Ermis Dionisios Geladas, Apostolos Spyros, Hao Xie, Georgios Tsiotis

**Affiliations:** 1 Department of Chemistry, University of Crete, P.O. Box 2208, GR-71003 Voutes, Greece; ilianakanavaki@gmail.com (I.K.); athinadrakonaki_@hotmail.com (A.D.); ermisgeladas@gmail.com (E.D.G.); aspyros@uoc.gr (A.S.); 2 Max Planck Institute of Biophysics, Max-von-Laue Strasse 3, D-60438 Frankfurt am Main, Germany

**Keywords:** *Pseudomonas* sp. strain phDV1, biodegradation, phenol, meta-cleavage pathway, biodegradable plastics, polyhydroxyalkanoates, poly(R)-hydroxyalkanoic acid synthase, class I

## Abstract

*Pseudomonas* strains have a variety of potential uses in bioremediation and biosynthesis of biodegradable plastics. *Pseudomonas* sp. strain phDV1, a Gram-negative phenol degrading bacterium, has been found to utilize monocyclic aromatic compounds as sole carbon source via the *meta*-cleavage pathway. The degradation of aromatic compounds comprises an important step in the removal of pollutants. The present study aimed to investigate the ability of the *Pseudomonas* sp. strain phDV1 to produce polyhydroxyalkanoates (PHAs) and examining the effect of phenol concentration on PHA production. The bacterium was cultivated in minimal medium supplemented with different concentrations of phenol ranging from 200–600 mg/L. The activity of the PHA synthase, the key enzyme which produces PHA, was monitored spectroscopically in cells extracts. Furthermore, the PHA synthase was identified by mass spectrometry in cell extracts analyzed by SDS-PAGE. Transmission electron micrographs revealed abundant electron-transparent intracellular granules. The isolated biopolymer was confirmed to be polyhydroxybutyrate (PHB) by FTIR, NMR and MALDI-TOF/TOF analyses. The ability of strain *Pseudomonas* sp. phDV1 to remove phenol and to produce PHB makes the strain a promising biocatalyst in bioremediation and biosynthesis of biodegradable plastics.

## 1. Introduction

Petroleum-derived plastic production and accumulation have devastating environmental effects, as approximately 99% of all non-degradable plastics are coming from the petrochemical market. Polyhydroxyalkanoates (PHAs) are bio-polyesters accumulated in cells by a wide range of bacteria under physiological stress [1]. PHAs are intracellular carbon and energy reserve materials. They are employed by microorganisms as a form of energy storage molecule in conditions such as limited availability of phosphorus or nitrogen [2]. PHAs appear to be true alternatives to common plastics because they are biodegradable and biocompatible as well as biologically produced [3]. However, the high cost of PHA is the limiting factor for its application as an alternative to traditional plastics [4]. Low-cost substrates, especially industrial waste, have attracted the attention of various researchers to reduce the PHA production cost [5].

Aromatic compounds are released into the environment as pollutants from industrial and urban activities. Phenol is highly toxic to all life forms in a wide range of concentrations (5–2000 mg/L) and is considered as a priority pollutant [6]. Microbial degradation is the most effective and economical way to remove aromatic pollutants, which is primarily accomplished by bacteria [7]. Studies have shown that phenol can be aerobically degraded by a wide variety of *Pseudomonas* species [8,9,10,11]. The *Pseudomonas* sp. strain phDV1 was isolated from an enriched mixed culture from samples of petroleum-contaminated soil in Denmark and its complete genome was recently reported [10,12]. At the genome level, 96.52% of the average nucleotide identity value (ANI) was obtained between *Pseudomonas* sp. phDV1 and *Pseudomonas pseudoalcaligenes* CECT534, suggesting that this strain belongs to the *Pseudomonas pseudoalcaligenes* species [12]. 

Strains of the *Pseudomonas* genus have become a focal point for studying the biodegradation of aromatic compounds. Mass spectrometry-based proteomics has been used to understand the aromatic biodegradation pathways in *Pseudomonas* sp. strain phDV1 [10,11,13,14,15]. These studies have shown that *Pseudomonas* sp. strain phDV1 can metabolize 2.2 mM phenol and 1.9 mM cresols, respectively, via the *meta*-cleavage pathway [10,13,15]. Recent studies show that monocyclic aromatic substrates influence the central carbon metabolism. This is reflected in the decreased abundance of glycolysis and gluconeogenesis enzymes and the increased abundance of the key enzymes of PHA synthesis [15]. Proteins involved in the synthesis of PHA, namely acetyl-CoA C-acetyltransferase, acetoacetyl-CoA reductase and poly-(3-hydroxyalkanoate) synthase, were found in high abundance in the presence of all monocyclic compounds [15]. The observed changes are consistent with the observed high abundance of the granule-associated proteins, namely the phasins [15]. Phenol is a toxic aromatic hydrocarbon solvent (partition coefficient log*P*ow = 1.5). To date, there have been no reports concerning the microbial biodegradation of phenol being subjected to improve PHA biosynthesis. PHA biosynthesis by *Pseudomonas* sp. strain phDV1 has unique elements that need further investigation. A very versatile *Pseudomonas* sp. strain phDV1 capable of utilizing phenol as carbon source for polymer production was reported in this study. It also demonstrates the potential of this strain for the production of copolymer poly (3-hydroxybutyrate) by a *meta*-cleavage pathway from totally unrelated carbon sources.

## 2. Materials and Methods

### 2.1. Cultivation of Pseudomonas sp. phDV1

The bacterium *Pseudomonas* sp. phDV1 was cultured as described in Tsirogiani et al. [13]. Briefly, cells were grown in M9 minimal medium at 32 °C and 200 rpm for 48 or 72 h. Phenol was added as the sole carbon source to the medium in different concentrations (200 mg/L, 400 mg/L and 600 mg/L). The growth was monitored by measuring the optical density using a UV2700 UV–vis spectrophotometer at 600 nm (Shimadzu, Kyoto, Japan). Consumption of phenol was monitored spectrophotometrically at 270 nm and the consumed amount was resupplied. The cells were collected at OD_600_ = 1.8−2 by centrifugation at 6000× *g* at 4 °C for 10 min. The pellet was washed twice with 50 mM Na_2_HPO_4_/NaH_2_PO_4_, pH 7.2 and then stored at −20 °C. All cultivations were performed in triplicates.

### 2.2. Activity Assay of PHA Synthase

Cell pellets were resuspended in 25 mM Tris-HCl (pH 7.5) containing 5% (*v*/*v*) glycerol in a ratio of 1 g cells per 5 mL buffer. Cells were broken by sonication with an Ultrasonicator Processor UP200 S (Hielscher, Teltow, Germany) with 30% amplitude, 1 cycle (20 times, 15 s with 45 s intervals), taking care to maintain the temperature below 10 °C. Unbroken cells and cell debris were removed from the resulting suspension by centrifugation at 13,000× *g* at 4 °C for 20 min and the supernatant was stored at −20 °C until further use. Assays of PHA synthase activity were carried out as described in literature with following modifications [16]. The assay mixtures (200 μL) contained 1.5 mM DL-b-hydroxybutyryl coenzyme A lithium salt (Santa Cruz Biotechnology, Dallas, Texas, USA ) and 0.5 mM 5, 5′-dithio-bis (2-nitrobenzoic acid) (DTNB, Sigma Aldrich, Darmstadt, Germany) in DMSO. The buffer solution in the reaction is 25 mM Tris-HCl with 5% glycerol, pH 7.5. The reaction was started by addition of 0.3 mg protein of the cell extracts. Absorption of the thiobenzoate anion resulting from the reaction of CoA and DTNB was measured at 412 nm for 10 min at room temperature using spectrophotometer (Multiscan Sky, Thermo Fisher, Dreieich, Germany). Protein concentration was determined by Bradford protein assay using bovine serum albumin as the standard (Multiscan Sky, Thermo Fisher, Dreieich, Germany).

### 2.3. SDS PAGE and MS-Based Protein Identification

20 μg protein of the cell extracts were analyzed by sodium dodecyl sulfate polyacrylamide gel electrophoresis (SDS-PAGE) using 4–12% Novex Tris-glycine gels (Invitrogen, Karlsruhe, Germany) and MOPS running buffer. Proteins were visualized by Coomassie blue staining. Protein bands of interest were excised from the gels and subjected to in-gel digestion (Thermo Scientific, Dreieich, Germany). The extracted peptides were analyzed by mass spectroscopy.

### 2.4. Transmission Electron Microscopy (TEM)

The PHA granules were observed with transmission electron microscopy. Samples from *Pseudomonas* sp. phDV1 grown in phenol for 72 h were centrifuged at 9000× *g* for 3 min and the cell pellet was fixed in a solution containing 2% (*w*/*v*) glutaraldehyde (GDA) and 2% (*w*/*v*) paraformaldehyde (PFA) in 100 mM sodium cacodylate (SCB) buffer (pH 7.4) for 90 min at room temperature. After washing twice with 4% sucrose and 0.1% SCB, cultures were post-fixed in 2% (*w*/*v*) osmium tetroxide (OsO_4_) at 4 °C for 60 min. After two washes, samples were dehydrated using 30, 50, 70, 85, 95, 100% (*v*/*v*) ethanol and were maintained at 4 °C to completely evaporate the ethanol. The samples were embedded in Durcupan A/M epoxy resin (Sigma-Aldrich, Darmstadt, Germany) and polymerized at 70 °C for 48 h. Thin sections of 70–100 nm were prepared using LKB 2088 Ultrotome V, stained with 2% (*w*/*v*) uranyl acetate, and finally samples were imaged using a JEM-2100 electron microscope operated at 80 kV.

### 2.5. Extraction of PHA 

After 72 h incubation, cells were harvested by centrifugation at 9000× *g* for 3 min. The cell pellet was weighed and suspended in hot chloroform at 60 °C for 15 min in a ratio of 1 g cells to 3 mL chloroform. The mixture was vortexed for 10 min in order to break the cells and the dissolved PHA was precipitated from chloroform by addition of four volumes of ice-cold methanol. PHA was obtained by centrifugation at 6000× *g* for 20 min and then dried at room temperature for 24 h. 

### 2.6. FTIR Analysis of the Purified PHA

Fourier-transform infrared (FTIR) analysis of PHA produced by *Pseudomonas* sp. phDV1 was performed using a Thermo-Electron Nicolet 6700 spectrophotometer. Sample was mixed well with KBr, and the absorbance of each sample was scanned from 400–4000 cm^−1^ with 128 scans.

### 2.7. NMR Spectroscopy of the Purified PHA 

The isolated PHA samples were dissolved in 600 μL of deuterated chloroform, CDCl_3_ and transferred in 5 mm NMR tubes after brief shaking. 1D and 2D NMR experiments were performed in either a Bruker DPX-300 or a Bruker Avance III 500 NMR spectrometer at a regulated temperature of 298 K, using standard Bruker pulse program libraries. 1D ^1^H NMR spectra were obtained with the following parameters: pulse program zg30, SW 20 ppm, AQ 3.3 s, TD 64 K, and ns 128. The homonuclear ^1^H-^1^H gradient-enhanced COSY 2D NMR spectrum was obtained with the following parameters: pulse program cosygpqf, 64 data points of 2Κ, ns 16, pulsed field gradients of 1 ms duration, and relaxation delay 1.5 s. Spectral processing and analysis were performed using TopSpin 4.0 (Bruker) software. All chemical shifts reported are referenced to the residual chloroform solvent peak (δ 7.26 ppm).

### 2.8. MALDI-TOF MS of the Purified PHA

The measurements were performed using a Rapiflex instrument (Bruker, Bremen, Germany). Isolated PHAs were solved in CHCl_3_ and mixed 1:1 with DHB (2,5-dihydroxybenzoic acid) matrix solution. 1 μL of the sample was spotted on a BigAnchor plate. Spectra were recorded in positive mode and in linear mode of 700–10,000 Da. Mass spectra measurements were performed applying the following criteria: smartbeam 3D laser and accumulation of 100,000 spectra in positive linear mode. Bruker Daltonics software “flexControl” was used for instrument operation and “flexAnalysis” for peak labeling.

## 3. Results and Discussion

### 3.1. Utilization of Phenol by Pseudomonas sp. phDV1

**Figure 1:**
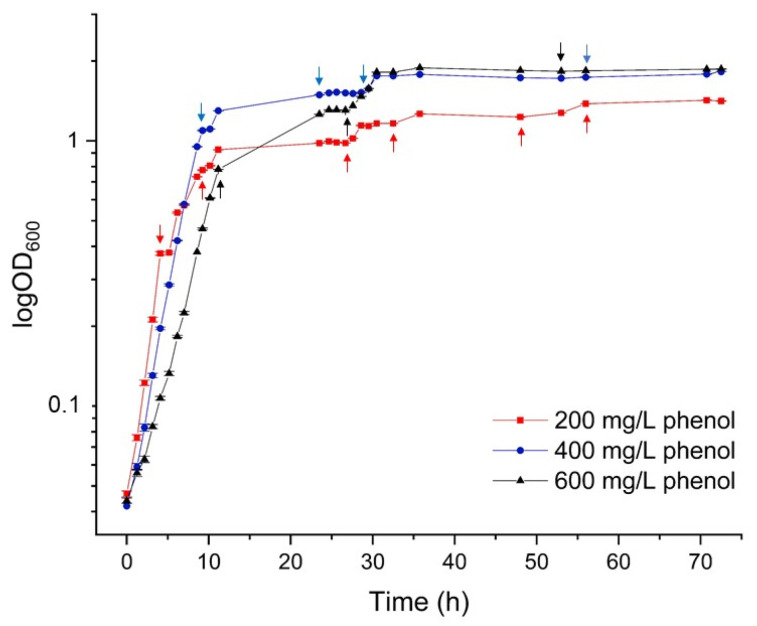
Growth curves of *Pseudomonas* sp. phDV1 under different concentrations of phenol. The arrows indicate the timepoints of phenol addition.

To investigate the effect of different concentrations of phenol (200, 400 and 600 mg/L) on bacterial growth, *Pseudomonas* sp. phDV1 was cultured in M9 minimal medium supplemented with phenol as sole carbon source. Cell growth was monitored by measuring OD at 600 nm for 72 h (Figure 1).

The results showed that *Pseudomonas* sp. phDV1 was able to degrade phenol in concentrations up to 600 mg/L. At 200 mg/L phenol, the bacterium showed a faster growth rate at the logarithmic phase and entered the early stationary phase after approximately 5 h of incubation. As the concentration of phenol increased, the log phase extended to 10 h and the slowest growth rate was observed with the concentration of 600 mg/L of phenol, which was probably due to increased toxicity arising from the use of phenol at this higher concentration. In addition, the maximum cell density was observed at the stationary phase in the presence of 600 mg/L of phenol. This observation suggests that, despite a slower growth rate, the highest PHA production level might be achieved by using 600 mg/L phenol since the elevated concentration of phenol provides more carbon and energy sources for cell mass production and PHA synthesis. 

**Figure 2:**
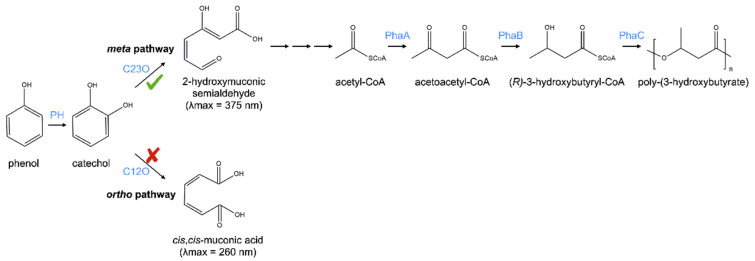
*Meta*-cleavage pathway of phenol degradation and the synthesis of PHA in *Pseudomonas* sp. phDV1. Abbreviations of enzymes: PH, phenol hydroxylase; C23O, catechol 2,3-dioxygenase; C12O, catechol 1,2-dioxygenase; PhaA, acetyl-CoA acetyltransferase; PhaB, acetyl-CoA reductase; PhaC, PHA synthase.

Our previous MS-based proteomics studies have shown that *Pseudomonas* sp. phDV1 can metabolize phenol via the *meta*-cleavage pathway of catechol degradation (Figure 2). When phenol was used as the sole carbon source, a bright yellow colouration was observed in the culture medium during the late phase of bacterial growth. This is a typical observation for the accumulation of 2-hydroxymuconic semialdehydes, which are produced from catechol by the catechol 2,3-dioxygenase [10,11,15]. To investigate whether the metabolic pathway can be altered by increasing the concentration of phenol, we investigated the catechol ring cleavage step by detecting the cleavage product in the whole-cell extracts. 

**Figure 3:**
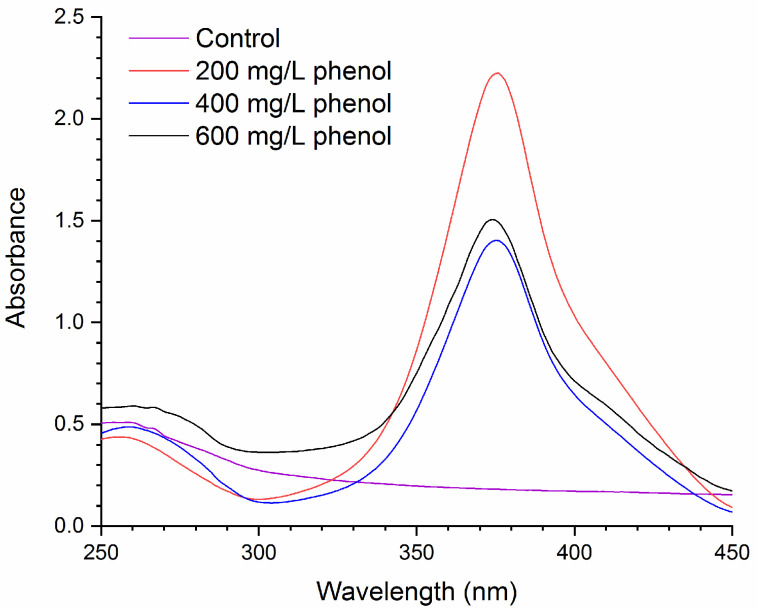
Detection of the catechol 2,3-dioxygenase activity in cell extracts of *Pseudomonas* sp. phDV1 grown under different concentrations of phenol. All sample spectra were recorded 6 min after the addition of catechol. Control spectrum was obtained in the absence of catechol.

Cell extracts were prepared from *Pseudomonas* sp. phDV1 grown in different phenol concentrations. The reaction was initiated by the addition of catechol and the absorption spectra in the range of 230–450 were recorded. As shown in Figure 3, after a 6 min incubation, a clear increase in absorbance at 375 nm was observed, indicating the presence of catechol 2,3-dioxygenase that initiates the *meta*-cleavage pathway of catechol degradation, whereas no significant change was observed at 260 nm, showing the absence of *ortho*-ring cleavage activity. Recently, the activation of both the *ortho*- and the *meta*-cleavage pathways has been reported in *Pseudomonas* *putida* P8 cells grown on a high benzoate concentration [17]. Our results clearly show that in *Pseudomonas* sp. phDV1 the high phenol concentration activated only the *meta*-cleavage catabolic pathway.

### 3.2. PHA Synthase Identification and Enzyme Assay

The genome of *Pseudomonas* sp. phDV1 contains three genes that potentially encode enzymes that catalyze the synthesis of PHA copolymers. Among them, one is annotated as the class I PHA synthase (UniProt ID: A0A385B2S5), while the second is classified as the class II PHA synthase (UniProt ID: A0A385B3I2). A third gene, which is predicted to encode another class II PHA synthase, was also found to be present. However, a frame shift is detected in its coding region probably due to a sequencing error (GenBank accession number: CP031606.1, locus tag: DZC76_02275) [12]. PHA synthase are grouped into four classes based on substrate specificity, and their preference in forming short-chain-length (scl) or medium-chain-length (mcl) polymers: Class I, Class III and Class IV produce principally scl-PHAs, while Class II PHA synthase synthesize mcl-PHAs [18,19]. A recent comprehensive analysis of the proteome of *Pseudomonas* sp. phDV1 using quantitative proteomics showed that monocyclic aromatic compounds influence the central carbon metabolism. This was directly reflected in the decreased abundance of glycolysis- and gluconeogenesis-related enzymes and the increased abundance of the key enzymes of PHA synthesis [15]. In addition, the class I PHA synthase can be identified by proteomic analysis when cells are grown on cresol or phenol, indicating that this enzyme may be involved in the PHA production [15]. To further investigate the PHA biosynthetic pathways in *Pseudomonas* sp. phDV1, whole-cell extracts were prepared from cells grown at different phenol concentrations and were analyzed for expression of PHA synthases by SDS-PAGE in combination with mass spectrometry (Supplementary Materials, Figure S1). Our results showed that only the class I PHA synthase can be detected, suggesting that in the presence of phenol; only this enzyme is employed to catalyze the polymerization of (R)-3-hydroxyacyl-CoA to PHA. *Pseudomonas* species are known to synthesize mainly mcl-PHAs, but some strains are able to synthesize both scl- and mcl-PHAs [20]. The detection of only the class I PHA synthase suggests that *Pseudomonas* sp. phDV1 cells synthesize principally scl-PHAs under phenol degradation conditions.

To confirm the presence of active PHA synthase, PHA synthase activity assays were performed using the whole-cell extracts by spectrometrically measuring the release of the coenzyme A during the polymerization process [16]. Starting from similar total-protein concentrations, higher concentration of the produced thiobenzoate anion was observed when cells were grown with 600 mg/L phenol, indicating a higher abundance of the PHA synthase (Table 1). 

### 3.3. Accumulation and Isolation of PHA 

**Figure 4:**
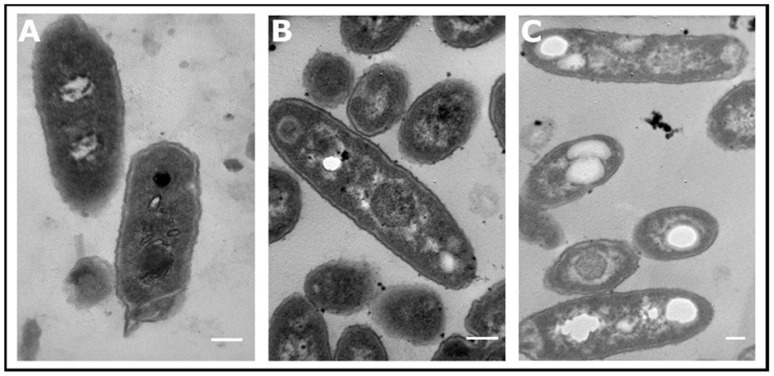
Transmission electron microscopy analyses of *Pseudomonas* sp. phDV1 cells grown for 72 h in M9 minimal medium in the presence of 200 mg/L (**A**), 400 mg/L (**B**) and 600 mg/L phenol (**C**). **A** and **B**: 20 k magnification; **C**: 12 k magnification. The bars correspond to 0.25 μm.

PHAs are accumulated as granular inclusions in cell cytoplasm that are typically 0.2–0.5 μm in diameter and can be visualized with different microscopical methods [21]. Recently, transmission electron microscopy (TEM) studies have been used to show the accumulation of white granules in *Pseudomonas* sp. strain phDV1 grown in 200 mL/L phenol as the carbon source [15]. To investigate if the formation of PHA granules upon increasing the phenol concentration was indeed feasible, we also analyzed cells for accumulated granules under the different growing conditions using TEM. In the presence of different phenol concentrations, *Pseudomonas* sp. phDV1 cells showed the ability to form electron transparent inclusion bodies (Figure 4). This is the first evidence that in the presence of relatively high concentrations of phenol (600 mg/L), *Pseudomonas* sp. phDV1 can synthesize this polymer. The granules have an average diameter of 0.1–0.2 μm which are slightly shorter than reported for many other PHA-producing bacteria. Nevertheless, PHA granules of similar size have been reported for *Pseudomonas mandelii* CBS-1 cultured aerobically with sucrose as the sole carbon source [22].

In addition, the produced PHAs were extracted from the cells by solvent extraction using chloroform and methanol. The highest yield of PHA, which was obtained when cells were cultured in the presence of 600 mg/L phenol, was 6.52 ± 0.21 mg of PHA per 1 g of wet cells. A yield of 4.64 ± 0.06 and 4.42 ± 0.91 mg of PHA per 1 g of wet cells was obtained for the conditions of 400 mg/L and 200 mg/L phenol concentration, respectively. These results are in correlation with our finding that the highest cell mass production and abundance of PHA synthase were attained when cultivation was conducted in the presence of 600 mg/L phenol.

### 3.4. Characterization of PHB by FTIR Analysis

**Figure 5:**
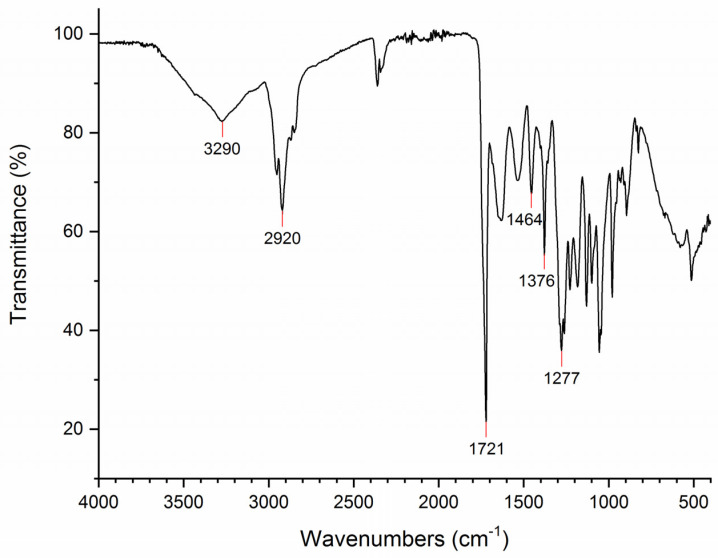
FTIR spectrum of isolated PHB produced by *Pseudomonas* sp. phDV1 grown in M9 minimal media supplemented with 600 mg/L phenol as the sole carbon source. The characteristic peaks associated with infrared-active functional groups of PHB are indicated by the red lines.

To characterize the PHA produced in *Pseudomonas* sp. phDV1, PHA polymers were isolated from cells grown in the presence of phenol and were analyzed by FTIR spectroscopy. In the presence of 600 mg/L phenol, the absorption peak at approximately 1277 cm^−1^ in the FTIR spectrum represents the saturated ester linkage of C–O groups (Figure 5). The absorption peaks at 1376 and 1464 cm^−1^ correspond to the respective stretching and bending mode of the vibration of the methyl (–CH_3_) group [23]. The absorption peaks at 1721, 2920 and 3290 cm^−1^ are the respective characteristic peaks of carbonyl (C = O), methine (-CH) and hydroxyl (-OH) groups, respectively [24,25]. The result of the FTIR analysis showed that the isolated polymer was polyhydroxybutyrate (PHB). Furthermore, the isolated PHA for the other two phenol concentrations showed all the characteristic peaks associated with PHB except the typical carbonyl peak [26]. The characteristic wide peak for the hydroxyl group located at 3434 cm^−1^ is overlapped by the presence of humidity in the sample [25]. 

### 3.5. Characterization of PHB by NMR Analysis 

**Figure 6:**
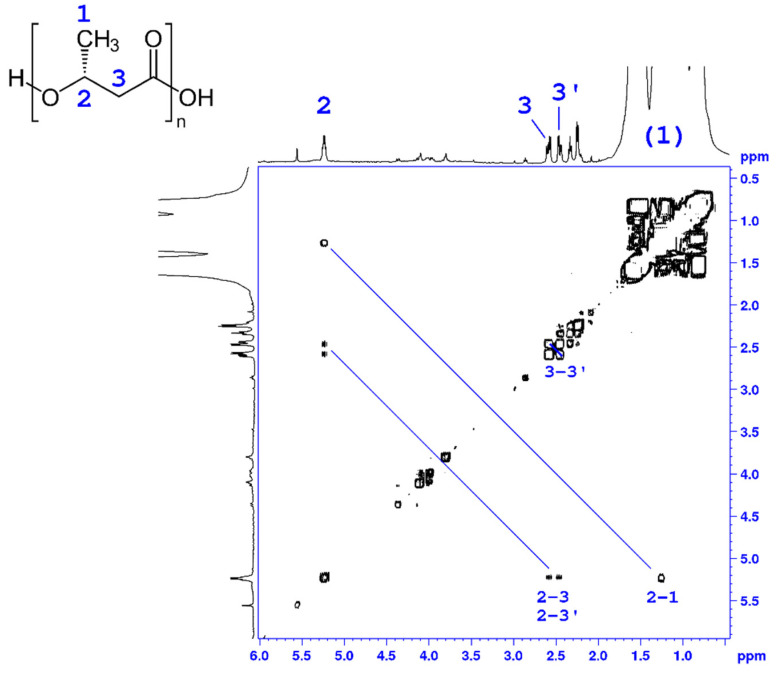
Homonuclear ^1^H-^1^H 2D gCOSY NMR spectrum of isolated PHB produced by *Pseudomonas* sp. phDV1 grown in M9 minimal media supplemented with 600 mg/L phenol as the sole carbon source. The spectrum was recorded in CDCl_3_ solution at 500.13 MHz.

In this work, ^1^H-NMR analysis was conducted to characterize the isolated polymers. The PHA polymers were extracted from cells grown under different concentrations of phenol (Figure 6, Supplementary Materials, Figure S2). The ^1^H NMR spectrum of the isolated PHA in CDCl_3_ solution (top projection in Figure 6) contains a signal at δ 5.26 (multiplet) and two doublets of doublets at δ 2.61 (15.5 and 7.4 Hz) and 2.47 (15.5 and 5.6 Hz), with integral ratios of 1:1:1. These ^1^H NMR spectral data (chemical shift and scalar couplings) are identical with those reported for the methine (2) –CH and methylene (3,3′) –CH2 protons of PHB isolated from *Bacillus megaterium* in the same solvent and temperature [27]. The signal of the methyl (1) –CH_3_ group of PHB is expected at δ 1.27 ppm; however, the aliphatic spectral region of the ^1^H NMR spectrum is dominated by signals arising from impurities in the sample. To overcome this, we have recorded a 2D heteronuclear ^1^H-^1^H gCOSY NMR experiment of the isolated PHA material, depicted in Figure 6, which shows correlations between neighboring protons connected via scalar *J* couplings. In the gCOSY 2D NMR spectrum the methine proton (2) is clearly connected via J coupling not only with the two methylene protons (3,3′), as expected, but also with a signal at δ 1.27, which coincides exactly with the chemical shift reported for the methyl protons (1) of PHB [27,28]. 2D NMR analysis thus provides unambiguous evidence for the identification of the isolated PHA as poly-3-hydroxybutyrate, P3HB [28].

### 3.6. Characterization of PHB by MALDI-TOF MS Analysis 

**Figure 7:**
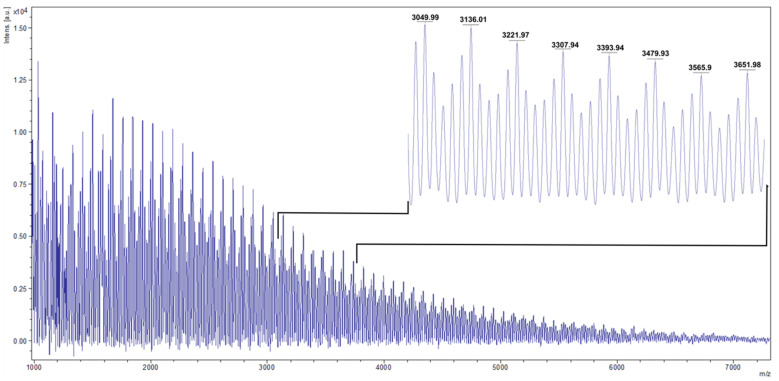
MALDI spectrum of isolated PHB produced by *Pseudomonas* sp. phDV1 grown in M9 minimal media supplemented with 600 mg/L phenol as the sole carbon source.

MALDI-TOF MS was used to perform end-group analysis to determine the mass of the isolated polymers [29]. The mass spectrum in the range of m/z = 800−7200 of the polymers isolated from cells grown in 600 mg/L phenol is shown in Figure 7. The oligomer was detected as MH^+^ adduct ion with a molecular weight of 86 Da. This obtained mass is in accordance with the expected molecular weight for the 3-hydroxybutyrate monomer (C_4_H_6_O_2_: 86 Da) and correlates well with our NMR results.

## 4. Conclusions

Bacterium *Pseudomonas* sp. phDV1 can utilize phenol as a sole carbon source and convert phenol into useful products. The structure of the produced PHA was analyzed by FTIR, NMR and MALDI MS. SDS-PAGE analysis showed that the class I PHA synthase is involved in the synthesis of PHA polymers under phenol cultivation conditions. Cell extracts with a higher phenol concentration show the highest PHA yield, in agreement with the observed higher abundance of the PHA synthase. These findings suggest the possibility of feeding the *Pseudomonas* sp. phDV1 with industrial pollutants to produce PHAs. In the present study, *Pseudomonas* sp. phDV1 was identified as a new PHB producer and our data provide the first biochemical framework for the optimization of PHB production in this bacterium.

## Figures and Tables

**Table 1:** PHA synthase activity assay. Absorption of the thiobenzoate anion resulting from the reaction of CoA and DTNB was measured at 412 nm and at 22 °C or 32 °C.

Phenol (mg/L)	A_412_ (22 °C)	TNB (mM)	A_412_ (32 °C)	TNB (mM)
200	0.366	0.0197	0.499	0.026
400	0.181	0.0097	0.426	0.023
600	0.396	0.0213	0.708	0.038

## Data Availability

Not applicable.

## Supplementary Materials

The following are available online at https://www.mdpi.com/article/10.3390/microorganisms9081636/s1. Figure S1. SDS-PAGE analysis of crude protein extracts isolated from *Pseudomonas* sp. strain phDV1 grown in 200 mg/L phenol (Lane 1), 400 mg/L phenol (Lane 2) and 600 mg/L phenol (Lane 3). Arrows indicate the protein bands in which the class I poly(R)-hydroxyalkanoic acid synthase (A0A385B2S5) was identified by mass spectroscopy. Figure S2. ^1^NMR Spectra of the isolated PHB from cell grown in 200 mg/L, 400 mg/L and 600 mg/L phenol.
