# Supplementary material for: Polyhydroxyalkanoate (PHA) Production in Pseudomonas sp. phDV1 Strain Grown on Phenol as Carbon Sources
Source: Microorganisms. 2021 Jul 30;9(8):1636. doi: 10.3390/microorganisms9081636 (PMC8398824; doi:10.3390/microorganisms9081636)
Supplement: Supplementary file 1 [file microorganisms-09-01636-s001.zip › microorganisms-1298172-supplementary.pdf]

Supplementary Materials

Polyhydroxyalkanoate (PHA) production in *Pseudomonas sp.* strain phDV1 grown on phenol as carbon source

Iliana Kanavaki[1], Athina Drakonaki[1], Ermis D. Geladas[1], Apostolos Spyros[1], Hao Xie[2] and Georgios Tsiotis[1]

1) Department of Chemistry, University of Crete, P.O. Box 2208, GR-71003 Voutes, Greece
2) Max Planck Institute of Biophysics, Max-von-Laue-Strasse 3, D-60438 Frankfurt am Main, Germany

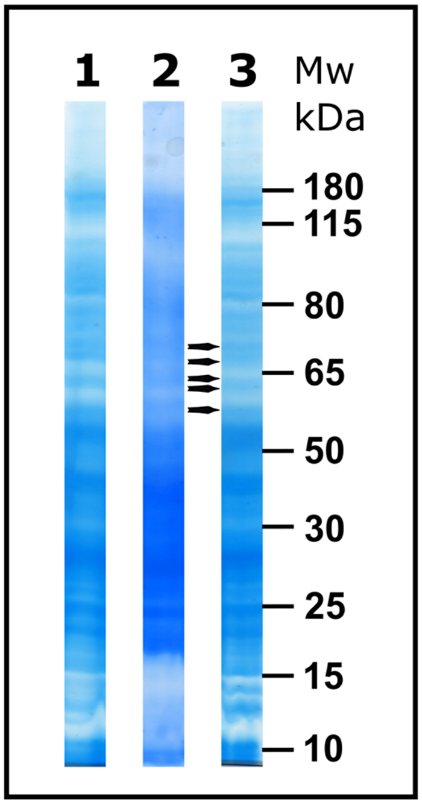

Figure S1. SDS-PAGE analysis of crude protein extracts isolated from *Pseudomonas* sp. strain phDV1 grown in 200 mg/L phenol (Lane 1), 400 mg/L phenol (Lane 2) and 600 mg/L phenol (Lane 3). Arrows indicate the protein bands in which the class I poly(R)-hydroxyalkanoic acid synthase (A0A385B2S5) was identified by mass spectroscopy.

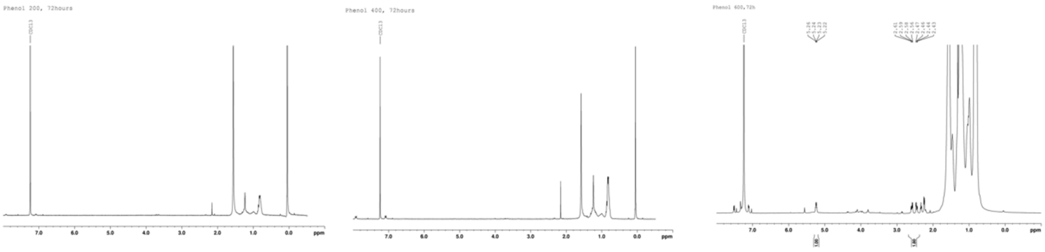

Figure S2. ^{1}NMR Spectra of the isolated PHB from cell grown in 200 mg/L, 400 mg/L and 600 mg/L phenol.
